# Crystal structure and synthesis of 3-(1*H*-pyrrol-2-yl)-1-(thio­phen-2-yl)propanone

**DOI:** 10.1107/S2056989018012331

**Published:** 2018-09-21

**Authors:** Dáire Gibbons, Ganapathi Emandi, Mathias O. Senge

**Affiliations:** a School of Chemistry, Trinity Biomedical Sciences Institute, 152–160 Pearse Street, Trinity College Dublin, The University of Dublin, Dublin 2, Ireland

**Keywords:** crystal structure, thio­phene, pyrrole, BODIPY, aza-BODIPY

## Abstract

The title compound, C_11_H_9_NOS, was obtained in an improved yield compared to previous literature methods. The mol­ecule is essentially planar with a maximum deviation of 0.085 Å from the mean plane through all non-H atoms. There is directive inter­molecular hydrogen bonding in the form of N—H⋯O hydrogen bonds with a distance of 2.889 (3) Å between the pyrrole amine and the ketone carbonyl O atom. The resulting hydrogen-bonding network defines a ribbon parallel to the *a* axis. These ribbons form offset stacks along the *b* axis.

## Abstract 2

The mol­ecule is essentially planar with a maximum deviation of 0.085 Å from the mean plane through all non-H atoms. In the crystal, N—H⋯O hydrogen bonds involving the pyrrole amine and the ketone carbonyl O atoms link the mol­ecules into [100] ribbons which form offset stacks along the *b* axis.

## Chemical context   

In nature, pyrroles are often present in tetra­pyrrolic ring systems such as heme and chloro­phyll. These macrocyclic compounds carry out a multitude of biochemical reactions and are responsible for oxygen transport in the body and harvesting light for food production in plants, respectively. Pyrroles are also widely incorporated in drugs, catalysts and advanced materials (Michlik & Kempe, 2013 ▸; Estévez *et al.*, 2014 ▸). The incorporation of pyrroles and thio­phenes into boron-dipyrromethene (BODIPY) dyes creates the possibility of long-wavelength absorptions and emissions (Schmidt *et al.*, 2009 ▸; Zrig *et al.*, 2008 ▸; Collado *et al.*, 2011 ▸; Rihn *et al.*, 2009 ▸; Gresser *et al.*, 2011 ▸; Ulrich *et al.*, 2007 ▸; Benniston *et al.*, 2008 ▸; Goeb & Ziessel, 2008 ▸). BODIPYs continue to be studied for their potential in fluorescence sensors, photodynamic therapy (PDT) and dye-sensitized solar cells (DSSCs) (Callaghan & Senge, 2018 ▸; Filatov *et al.*, 2018 ▸; Boens *et al.*, 2011 ▸, 2012 ▸; Antina *et al.*, 2017 ▸; Kamkaew *et al.*, 2013 ▸; Singh & Gayathri, 2014 ▸; Loudet & Burgess, 2007 ▸; Er *et al.*, 2015 ▸; Kand *et al.*, 2015 ▸; Cheng *et al.*, 2016 ▸). Changing the *meso-*carbon of the BODIPY to a nitro­gen atom creates an aza-BODIPY compound. The absorption and emission in an aza-BODIPY is shifted more towards the near infra-red region compared to BODIPY (Lu *et al.*, 2014 ▸; Balsukuri, *et al.*, 2018 ▸). Herein, we report the improved synthesis and crystal structure of a previously synthesized ketone (Stark *et al.*, 2016 ▸) that can be further functionalized to create a sophisticated aza-BODIPY.
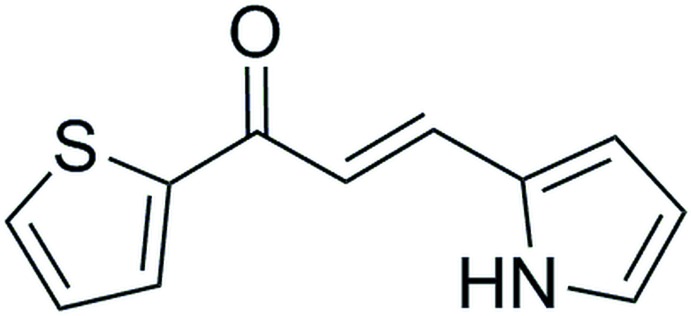


## Structural commentary   

**Figure 1:**
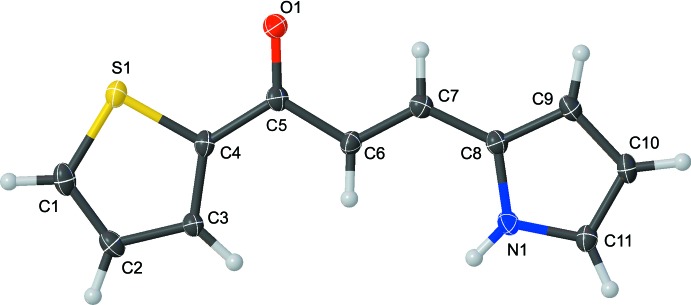
The mol­ecular structure of the title compound, showing the atom labelling. Displacement ellipsoids are drawn at the 50% probability level.

**Figure 2:**
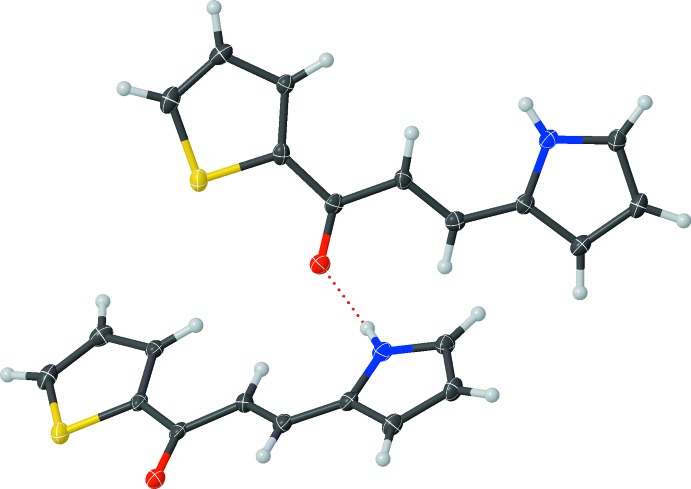
The hydrogen bonding (dashed line) between the amine group and the carbonyl oxygen atom (Table 1 ▸). Displacement ellipsoids are drawn at the 50% probability level.

The title compound **1** crystallizes in a polar non-centrosymmetric space group (*Pna*2_1_) and is almost planar in its crystalline form (Fig. 1 ▸; Table 1 ▸) with deviations ranging from −0.059 (3) (C11) to 0.085 Å (C7) from the mean plane of all non-hydrogen atoms. The pyrrole ring (N1/C8–C11) is rotated out of the plane through the ketone and thio­phenyl groups (S1/O1/C1–C7) by 4.32 (10)°. The aliphatic chain linking the two ring systems has a *trans* conformation and the nitro­gen atom (N1) of the pyrrole ring is protonated. Atom N1 is oriented opposite to the sulfur atom S1 of the thio­phene ring to enable inter­molecular hydrogen bonding (Fig. 2 ▸). Atom S1 lies on the same side of the mol­ecular backbone as the oxygen of the ketone (O1).

## Supra­molecular features   

**Figure 3:**
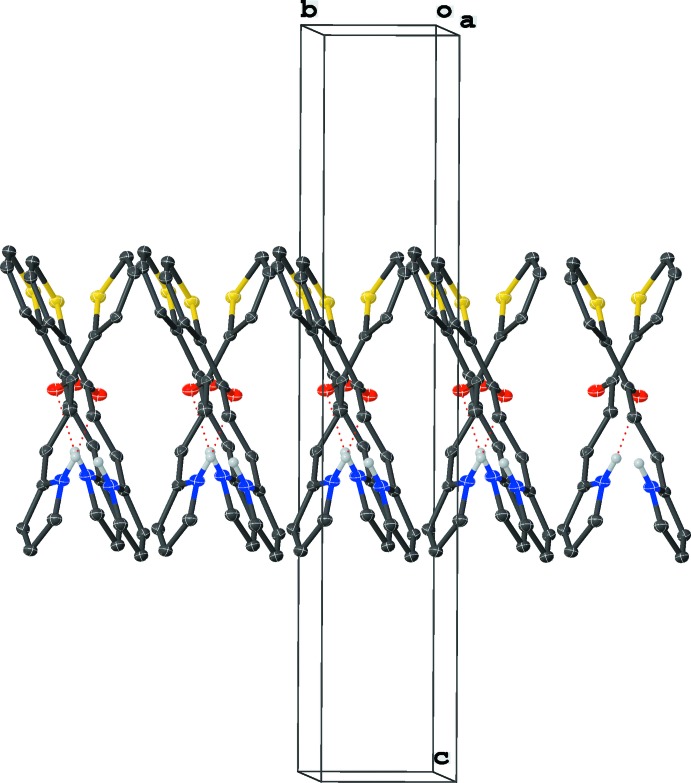
View of the X-pattern in the packing, viewed approximately along the *a* axis. Displacement ellipsoids are drawn at the 50% probability level.

Hydrogen bonding dominates the crystal packing of **1** and occurs between the amine group and the carbonyl oxygen (Fig. 2 ▸, Table 1 ▸), linking the mol­ecules into a head-to-head ribbon-type assembly that extends down the *a* axis in an alternating X-pattern (Fig. 3 ▸). The angle between the alternating mol­ecules in this X-pattern is 88.804 (8)°. The ribbons form offset stacks along the *b* axis with centroid–centroid distances of 3.9257 (15) Å between the centroids of adjacent pyrrole or thio­phene rings and a plane shift distance of 1.89 (3) Å between any two mol­ecules in the three-dimensional crystal structure. C—H⋯O inter­actions also occur.

## Database survey   

A search of the Cambridge Structural Database (CSD, version 5.39; Groom *et al.*, 2016 ▸) gave two structures of aza-BODIPY precursor derivatives of **1** (Table 2 ▸). In (*E*)-1,3-di(thio­phen-2-yl)prop-2-en-1-one (LINFET; Li & Su, 1995 ▸), a thio­phene ring replaces the pyrrole ring, yielding a di-thio­phene-linked α,β-unsaturated ketone aliphatic chain. LINFET contains two independent mol­ecules in the asymmetric unit. One di­thio­phene-linked chain is less planar than in **1** (LINFET A) while the other is more planar (LINFET B; Table 2 ▸). The deviations from the plane of LINFET A range from −0.127 Å (C5) to 0.233 Å (S1); the deviations in LINFET B are smaller ranging from −0.032 Å for C22 to 0.055 Å for O2. They both exhibit the same *trans* conformation seen in the title mol­ecule. Non-classical hydrogen bonding exists between a C—H group and the carbonyl oxygen, O1 with a C—H⋯O distance of 3.326 (6) Å. This bonding network results in three separate sheets, parallel to the *a-*axis. A second non-classical hydrogen-bonding network [C—H⋯O = 2.381 (4) Å] is observed extending along the *c-*axis direction, generating a staggered ribbon. The combination of these two networks gives rise to a three-dimensional structure.

(*E*)-1,3-Di(furan-2-yl)prop-2-en-1-one (SANRIJ; Ocak Ískeleli *et al.*, 2005 ▸) comprises two furan heterocycles linked by an α,β-unsaturated ketone aliphatic chain. There are also two independent mol­ecules in the asymmetric unit (SANRIJ A and SANRIJ B), both of which are less planar than **1**, LINFET A and LINFET B. The largest deviation from the mol­ecular plane is for C17 (0.157 Å) in SANRIJ A and C18 (−0.152 Å) in SANRIJ B. Again, a non-classical hydrogen bonding network exists [C—H⋯O = 2.473 (18) Å] between aryl C—H atoms and the carbonyl oxygen. Each mol­ecule participates in two hydrogen bonds and the network extends in a linear fashion along the *b-*axis direction, forming a network structure.

## Synthesis and crystallization   

The title compound was synthesized *via* an elimination unimolecular conjugate base (E1cB) reaction between 2-pyrrole-carbaldehyde (376.87 mg, 3.96 mmol, 1.0 eq.) and 2-acetyl­thio­phene (500 mg, 3.96 mmol, 1.0 eq.) in 1:1 MeOH:H_2_O (10 ml) using NaOH (15.85 mg, 396.28 µmol, 0.1 eq.) as a base. The resulting precipitate was filtered and then crystallized using a solution of CHCl_3_, layered with hexane to give a single crystal suitable for X-ray diffraction. [C_11_H_9_NOS]: yield 85% m.p 420–430 K.

^1^H NMR (CDCl_3_, ppm, 400MHz): δ 6.34 (*dd*, *J* = 5.9, 2.6 Hz, 1H, =C—H), 6.74 (*s*, 1H, Ar-H), 7.01 (*s*, 1H, Ar-H), 7.06–7.10 (*d*, 1H, Ar-H), 7.15 (*t*, *J* = 8.7 Hz, 1H, Ar-H), 7.63 (*d*, *J* = 4.9 Hz, 1H, Ar-H), 7.81 (*t*, 1H, Ar-H), 7.83 (*d*, *J* = 10.3 Hz, 1H, =C—H), 9.17 (*s*, 1H, NH). ^13^C NMR (CDCl_3_, ppm, 400 MHz): δ 111.54, 115.16, 123.30, 128.15, 129.16, 131.11, 133.17, 133.98, 145.89, 182.05. HRMS (ESI): *m*/*z* calculated for C_11_H_9_NOS: 204.047690 (*M* + H)^+^. Found: 204.04776.

## Refinement   

Crystal data, data collection and structure refinement details are summarized in Table 3 ▸. H atoms were placed in their expected calculated positions and refined as riding: C—H = 0.95–0.98 Å with *U*_iso_(H) = 1.2 *U*_eq_(C).

## Supplementary Material

Crystal structure: contains datablock(s) I. DOI: 10.1107/S2056989018012331/tx2008sup1.cif

Structure factors: contains datablock(s) I. DOI: 10.1107/S2056989018012331/tx2008Isup2.hkl

Supporting information file. DOI: 10.1107/S2056989018012331/tx2008Isup3.cml

CCDC reference: 1864793

Additional supporting information:  crystallographic information; 3D view; checkCIF report

## Figures and Tables

**Table 1:** Hydrogen-bond geometry (Å, °)

*D*—H⋯*A*	*D*—H	H⋯*A*	*D*⋯*A*	*D*—H⋯*A*
N1—H1*A*⋯O1^i^	0.84 (4)	2.06 (4)	2.889 (3)	171 (3)
C6—H6⋯O1^i^	0.95	2.50	3.396 (3)	158

Symmetry code: (i) 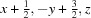.

**Table 2:** r. m. s. deviations and twist (Å, °) angles The twist angle is the dihedral angle between the five-membered heterocycle and the keto-aromatic plane.

Compound	r.m.s. deviation	Twist angle
**1**	0.04 (8)	4.32 (10)
LINFET A^*a*^	0.111 (2)	10.21 (12)
LINFET B^*a*^	0.023 (2)	1.19 (15)
SANRIJ A^*b*^	0.104 (15)	8.98 (4)
SANRIJ B^*b*^	0.122 (20)	9.67 (6)

Notes: (*a*) Li & Su (1995); (*b*) Ocak Ískeleli *et al.* (2005).

**Table 3:** Experimental details

Crystal data	
Chemical formula	C_11_H_9_NOS
*M*_r_	203.25
Crystal system, space group	Orthorhombic, *P* *n* *a*2_1_
Temperature (K)	100
*a*, *b*, *c* (Å)	11.1559 (3), 3.9258 (1), 21.6293 (6)
*V* (Å^3^)	947.27 (4)
*Z*	4
Radiation type	Mo *K*α
μ (mm^−1^)	0.30
Crystal size (mm)	0.2 × 0.09 × 0.07

Data collection	
Diffractometer	Bruker SMART APEXII area detector
Absorption correction	Multi-scan (*SADABS*; Krause *et al.*, 2015 ▸)
*T*_min_, *T*_max_	0.658, 0.746
No. of measured, independent and observed [*I* > 2σ(*I*)] reflections	25996, 2159, 2070
*R*_int_	0.049
(sin θ/λ)_max_ (Å^−1^)	0.650

Refinement	
*R*[*F* ^2^ > 2σ(*F* ^2^)], *wR*(*F* ^2^), *S*	0.032, 0.082, 1.06
No. of reflections	2159
No. of parameters	131
No. of restraints	1
H-atom treatment	H atoms treated by a mixture of independent and constrained refinement
Δρ_max_, Δρ_min_ (e Å^−3^)	0.28, −0.17
Absolute structure	Flack *x* determined using 956 quotients [(*I* ^+^)−(*I* ^−^)]/[(*I* ^+^)+(*I* ^−^)] (Parsons *et al.*, 2013 ▸)
Absolute structure parameter	0.00 (4)

Computer programs: *APEX3* and *SAINT* (Bruker, 2016 ▸), *SHELXT* (Sheldrick, 2015*a* ▸), *SHELXL* (Sheldrick, 2015*b* ▸) and *OLEX2* (Dolomanov *et al.*, 2009 ▸).

## supplementary crystallographic information

### Crystal data

**Table:**

C_11_H_9_NOS	*D*_x_ = 1.425 Mg m^−^^3^
*M**_r_* = 203.25	Mo *K*α radiation, λ = 0.71073 Å
Orthorhombic, *P**n**a*2_1_	Cell parameters from 9918 reflections
*a* = 11.1559 (3) Å	θ = 3.7–27.5°
*b* = 3.9258 (1) Å	µ = 0.30 mm^−^^1^
*c* = 21.6293 (6) Å	*T* = 100 K
*V* = 947.27 (4) Å^3^	Triangular, yellow
*Z* = 4	0.2 × 0.09 × 0.07 mm
*F*(000) = 424	

### Data collection

**Table:**

Bruker SMART APEXII area detector diffractometer	2159 independent reflections
Radiation source: standard sealed X-ray tube, Siemens, KFF Mo 2K -90 C	2070 reflections with *I* > 2σ(*I*)
Graphite monochromator	*R*_int_ = 0.049
Detector resolution: 7.9 pixels mm^-1^	θ_max_ = 27.5°, θ_min_ = 1.9°
ω and φ scans	*h* = −14→14
Absorption correction: multi-scan (SADABS; Krause *et al.*, 2015)	*k* = −5→5
*T*_min_ = 0.658, *T*_max_ = 0.746	*l* = −27→28
25996 measured reflections	

### Refinement

**Table:**

Refinement on *F*^2^	Hydrogen site location: mixed
Least-squares matrix: full	H atoms treated by a mixture of independent and constrained refinement
*R*[*F*^2^ > 2σ(*F*^2^)] = 0.032	*w* = 1/[σ^2^(*F*_o_^2^) + (0.0533*P*)^2^ + 0.3056*P*] where *P* = (*F*_o_^2^ + 2*F*_c_^2^)/3
*wR*(*F*^2^) = 0.082	(Δ/σ)_max_ < 0.001
*S* = 1.06	Δρ_max_ = 0.28 e Å^−^^3^
2159 reflections	Δρ_min_ = −0.17 e Å^−^^3^
131 parameters	Absolute structure: Flack *x* determined using 956 quotients [(*I*^+^)-(*I*^-^)]/[(*I*^+^)+(*I*^-^)] (Parsons *et al.*, 2013)
1 restraint	Absolute structure parameter: 0.00 (4)
Primary atom site location: dual	

### Special details

**Table:**

Geometry. All esds (except the esd in the dihedral angle between two l.s. planes) are estimated using the full covariance matrix. The cell esds are taken into account individually in the estimation of esds in distances, angles and torsion angles; correlations between esds in cell parameters are only used when they are defined by crystal symmetry. An approximate (isotropic) treatment of cell esds is used for estimating esds involving l.s. planes.

### Fractional atomic coordinates and isotropic or equivalent isotropic displacement parameters (Å^2^)

**Table:**

	*x*	*y*	*z*	*U*_iso_*/*U*_eq_	
C1	0.4383 (3)	0.3593 (7)	0.30581 (13)	0.0220 (6)	
H1	0.4090	0.2839	0.2669	0.026*	
C2	0.5545 (3)	0.3241 (7)	0.32434 (13)	0.0199 (5)	
H2	0.6149	0.2208	0.2997	0.024*	
C3	0.5754 (2)	0.4580 (6)	0.38425 (12)	0.0146 (5)	
H3	0.6510	0.4546	0.4045	0.018*	
C4	0.4712 (2)	0.5956 (7)	0.40983 (12)	0.0154 (5)	
C5	0.4521 (2)	0.7623 (7)	0.46958 (11)	0.0158 (5)	
C6	0.5566 (2)	0.7940 (7)	0.51008 (13)	0.0167 (5)	
H6	0.6303	0.6917	0.4983	0.020*	
C7	0.5498 (2)	0.9663 (7)	0.56396 (12)	0.0166 (5)	
H7	0.4753	1.0719	0.5733	0.020*	
C8	0.6436 (2)	1.0055 (7)	0.60829 (13)	0.0160 (5)	
C9	0.6393 (2)	1.1517 (7)	0.66686 (12)	0.0183 (5)	
H9	0.5713	1.2570	0.6852	0.022*	
C10	0.7529 (3)	1.1168 (7)	0.69428 (12)	0.0195 (5)	
H10	0.7758	1.1928	0.7343	0.023*	
C11	0.8247 (2)	0.9509 (7)	0.65194 (13)	0.0182 (6)	
H11	0.9065	0.8924	0.6579	0.022*	
N1	0.7588 (2)	0.8850 (6)	0.60020 (11)	0.0179 (5)	
H1A	0.787 (3)	0.791 (9)	0.5687 (16)	0.019 (8)*	
O1	0.35144 (16)	0.8661 (6)	0.48409 (9)	0.0199 (4)	
S1	0.35151 (5)	0.55340 (15)	0.36015 (3)	0.01928 (17)	

### Atomic displacement parameters (Å^2^)

**Table:**

	*U*^11^	*U*^22^	*U*^33^	*U*^12^	*U*^13^	*U*^23^
C1	0.0338 (15)	0.0180 (12)	0.0144 (12)	−0.0026 (11)	−0.0025 (10)	0.0009 (10)
C2	0.0266 (13)	0.0172 (13)	0.0160 (12)	−0.0001 (10)	0.0048 (10)	−0.0005 (11)
C3	0.0173 (11)	0.0139 (11)	0.0126 (11)	−0.0020 (9)	0.0005 (9)	0.0009 (9)
C4	0.0187 (12)	0.0154 (12)	0.0120 (11)	−0.0046 (9)	−0.0014 (9)	0.0023 (9)
C5	0.0200 (12)	0.0166 (10)	0.0108 (11)	−0.0016 (10)	0.0010 (10)	0.0036 (10)
C6	0.0158 (11)	0.0201 (12)	0.0140 (11)	−0.0006 (10)	−0.0014 (10)	0.0010 (11)
C7	0.0188 (12)	0.0165 (12)	0.0146 (12)	−0.0002 (10)	0.0013 (10)	0.0030 (10)
C8	0.0195 (12)	0.0155 (12)	0.0130 (12)	−0.0010 (10)	0.0007 (9)	0.0016 (9)
C9	0.0244 (13)	0.0168 (12)	0.0138 (12)	0.0011 (10)	0.0017 (10)	−0.0013 (10)
C10	0.0274 (13)	0.0182 (12)	0.0129 (12)	−0.0024 (10)	−0.0025 (10)	−0.0010 (10)
C11	0.0194 (13)	0.0205 (13)	0.0149 (12)	−0.0040 (10)	−0.0010 (10)	0.0006 (10)
N1	0.0191 (11)	0.0218 (11)	0.0129 (11)	−0.0012 (9)	0.0016 (9)	−0.0015 (9)
O1	0.0183 (9)	0.0272 (9)	0.0143 (9)	0.0008 (8)	0.0013 (7)	0.0014 (8)
S1	0.0198 (3)	0.0223 (3)	0.0157 (3)	−0.0019 (2)	−0.0033 (3)	0.0002 (3)

### Geometric parameters (Å, º)

**Table:**

C1—H1	0.9500	C6—C7	1.350 (4)
C1—C2	1.364 (4)	C7—H7	0.9500
C1—S1	1.702 (3)	C7—C8	1.428 (4)
C2—H2	0.9500	C8—C9	1.392 (4)
C2—C3	1.418 (4)	C8—N1	1.381 (3)
C3—H3	0.9500	C9—H9	0.9500
C3—C4	1.397 (4)	C9—C10	1.406 (4)
C4—C5	1.464 (3)	C10—H10	0.9500
C4—S1	1.722 (3)	C10—C11	1.380 (4)
C5—C6	1.463 (3)	C11—H11	0.9500
C5—O1	1.236 (3)	C11—N1	1.364 (4)
C6—H6	0.9500	N1—H1A	0.84 (4)
			
C2—C1—H1	123.7	C6—C7—C8	126.4 (3)
C2—C1—S1	112.5 (2)	C8—C7—H7	116.8
S1—C1—H1	123.7	C9—C8—C7	129.1 (2)
C1—C2—H2	123.6	N1—C8—C7	124.1 (2)
C1—C2—C3	112.8 (3)	N1—C8—C9	106.8 (2)
C3—C2—H2	123.6	C8—C9—H9	125.9
C2—C3—H3	124.2	C8—C9—C10	108.2 (2)
C4—C3—C2	111.6 (2)	C10—C9—H9	125.9
C4—C3—H3	124.2	C9—C10—H10	126.6
C3—C4—C5	130.0 (2)	C11—C10—C9	106.8 (2)
C3—C4—S1	111.2 (2)	C11—C10—H10	126.6
C5—C4—S1	118.77 (19)	C10—C11—H11	125.6
C6—C5—C4	116.8 (2)	N1—C11—C10	108.7 (2)
O1—C5—C4	120.2 (2)	N1—C11—H11	125.6
O1—C5—C6	122.9 (2)	C8—N1—H1A	127 (2)
C5—C6—H6	119.5	C11—N1—C8	109.4 (2)
C7—C6—C5	121.0 (2)	C11—N1—H1A	124 (2)
C7—C6—H6	119.5	C1—S1—C4	91.90 (14)
C6—C7—H7	116.8		
			
C1—C2—C3—C4	−0.2 (3)	C7—C8—C9—C10	177.3 (3)
C2—C1—S1—C4	0.4 (2)	C7—C8—N1—C11	−177.4 (3)
C2—C3—C4—C5	−179.2 (2)	C8—C9—C10—C11	0.2 (3)
C2—C3—C4—S1	0.5 (3)	C9—C8—N1—C11	0.3 (3)
C3—C4—C5—C6	0.0 (4)	C9—C10—C11—N1	0.0 (3)
C3—C4—C5—O1	−179.5 (3)	C10—C11—N1—C8	−0.2 (3)
C3—C4—S1—C1	−0.5 (2)	N1—C8—C9—C10	−0.3 (3)
C4—C5—C6—C7	175.0 (2)	O1—C5—C6—C7	−5.6 (4)
C5—C4—S1—C1	179.2 (2)	S1—C1—C2—C3	−0.2 (3)
C5—C6—C7—C8	177.7 (2)	S1—C4—C5—C6	−179.67 (19)
C6—C7—C8—C9	−173.4 (3)	S1—C4—C5—O1	0.9 (3)
C6—C7—C8—N1	3.8 (4)		

### Hydrogen-bond geometry (Å, º)

**Table:**

*D*—H···*A*	*D*—H	H···*A*	*D*···*A*	*D*—H···*A*
N1—H1*A*···O1^i^	0.84 (4)	2.06 (4)	2.889 (3)	171 (3)
C6—H6···O1^i^	0.95	2.50	3.396 (3)	158

Symmetry code: (i) *x*+1/2, −*y*+3/2, *z*.

### Route mean square (r. m. s.) deviations and twist angles of the two five-membered heterocycles in 1 and the two derivatives known in literature (Li &amp; Su, 1995; Ocak Ískeleli *et al.*, 2005)

**Table:**

Compound	R. M. S. deviation (Å)	Twist angle (o)
1	0.056	2.87 (11)
2 (First independent molecule)	0.093 (2)	7.15 (6)
Second independent molecule	0.02 (2)	0.20 (15)
3 (First independent molecule)	0.104	8.41 (6)
Second independent molecule	0.122	11.11 (7)
